# Volume-time curve of cardiac magnetic resonance assessed left ventricular dysfunction in coronary artery disease patients with type 2 diabetes mellitus

**DOI:** 10.1186/s12872-017-0583-5

**Published:** 2017-06-05

**Authors:** Hua-yan Xu, Zhi-gang Yang, Ying-kun Guo, Ke Shi, Xi Liu, Qin Zhang, Li Jiang, Lin-jun Xie

**Affiliations:** 1 Department of Radiology, National Key Laboratory of Biotherapy, West China Hospital, Sichuan University, # 37Guo Xue Xiang, Chengdu, Sichuan 610041 China; 2 Department of Radiology, West China Second University Hospital, Sichuan University, #20, Section 3, Renmin South Road, Chengdu, Sichuan 610041 China

## Abstract

**Background:**

Type 2 diabetes mellitus (DM2) may induce epicardial coronary artery diseases and left ventricular myocardial damaging as well. Left ventricular dysfunction was found in DM2. In this research, we compared the left ventricular dysfunction of coronary artery disease (CAD) patients with and without type 2 diabetes mellitus as well as normal controls using the volume-time curve of cardiac magnetic resonance (CMR).

**Methods:**

Sixty-one CAD patients (28 with DM2 and 33 without DM2) and 18 normal individuals were enrolled in this study. Left ventricular function parameters, including the end-diastolic and end-systolic volumes (EDV, ESV), stroke volume (SV) and ejection fraction (EF), and morphologic dimension parameters (end diastolic and systolic diameter (EDD and ESD), were measured and compared. Volume-time curve parameters, including the peak ejection rate (PER), peak ejection time (PET), peak filling rate (PFR), peak filling time from ES (PFT), peak ejection rate normalized to EDV (PER/EDV), and peak filling rate normalized to EDV (PFR/EDV), were derived automatically and compared.

**Results:**

LVEF in the diabetic CAD group was markedly reduced when compared to the normal and CAD without DM2 groups (all *p* < 0.05). LVEDD of the diabetic CAD group was significantly enlarged compared to the normal and non-diabetic CAD groups (all *p* < 0.05). More importantly, the lowest parameters of the left ventricle volume time curve (i.e., PER, PFR, PER/EDV and PFR/EDV) were obtained in diabetic CAD patients (all *p* < 0.05). In diabetic CAD patients, logistic regression analysis indicated that PET, PFT and PFR/EDV were independent predictors of left ventricular dysfunction (odds ratio [OR]: 1.1208, 1.0161, and 0.0139, respectively). The sensitivity and specificity of PET were 81.2 and 90%, respectively, when the threshold value was greater than 164.4 msec; for PFT, the sensitivity and specificity were 87.5 and 95.0%, respectively (criterion >166.0 msec). Higher sensitivity (87.5%) and specificity (100.0%) were obtained for PFR/EDV (criterion ≤3.7EDV/s).

**Conclusions:**

Parameters that are derived from the volume-time curve on CMR, including PET, PFT and PFR/EDV, allow clinicians to predict left ventricular dysfunction in diabetic CAD subjects with a high degree of sensitivity and specificity.

## Background

Diabetes mellitus (DM) is a chronic metabolic disease in which the body cannot produce enough insulin or effectively utilize it. Recently, there has been an increase in the number of DM patients worldwide. World Health Organization (WHO) predicts that number of DM patients will reach 552 million by 2030 [1]. In patients with type 2 diabetes mellitus (DM2), the risk of cardiovascular diseases, such as coronary artery disease (CAD), is approximately double compared to non-diabetics [2–4]. Furthermore, the long-term incidence of cardiac death (i.e., myocardial ischemia) or worse long-term outcomes was higher in diabetic patients [5, 6]. Left ventricular dysfunction has been demonstrated in approximately 43–75% of DM2 patients; furthermore, this dysfunction has been associated with increased mortality and heart failure [7, 8]. A better understanding of the underlying effects of DM2 on CAD with regard to cardiac function may improve our therapeutic efforts and lengthen patients’ lives. However, reports that examine left ventricular dysfunction in CAD patients with DM2 are limited. Moreover, differences in cardiac function and remodeling among normal group, CAD patients with and without DM2 need to be demonstrated further.

Cardiac magnetic resonance (CMR) is considered to be the gold standard modality for estimating global left ventricular function because it is noninvasive and yields high soft-tissue contrast and temporal resolution [9, 10]. Currently, the volume-time curve is a promising metric that can be used to evaluate continuous volume changes in the left ventricle. Its parameters include peak ejection rate (PER), peak ejection time (PET), peak filling rate (PFR), peak filling time from ES (PFT), peak ejection rate normalized to EDV (PER/EDV), and peak filling rate normalized to EDV (PFR/EDV). Among these volume-time curve parameters, PER, PET and PER/EDV are the indices of systolic function; the other parameters represent diastolic function [11]. In clinical practice, the volume-time curve is generally accepted to be a crucial supplement to routine cardiac function measurement. Previous studies have indicated that PFR is reduced and PFT is increased in coronary artery disease patients [12]. Meanwhile, the diabetes mellitus is a major risk factor for the development of LV dysfunction even in the absence of symptom of, and the T2DM have an evidence of systolic ventricular dysfunction [13]. In addition, Graça et cal found that patients with DM and coronary atherosclerosis show a more impaired LV diastolic function than patients without coronary atherosclerosis by only using LV PFR normalized to stroke volume of CMR [14]. However, to the best of our knowledge, detailed global LV function assessed by in CAD patients with DM2 compared with CAD without DM2 is currently insufficient and certain LV diastolic and systolic function parameters that are derived from the volume-time curve are still not comprehensive. Thus, the specific aim of our study was to investigate whether the volume-time curve on cardiac magnetic resonance is able to assess and differentiate the LV diastolic and systolic dysfunction in CAD patients with/without type 2 diabetes comparing with normal controls.

## Methods

### Study population

During December 2014 and August 2015, 28 CAD with DM2 (18 males and 10 females; mean age, 58.12 ± 12.53 yrs.; age range, 30-78 yrs) and 33 CAD patients without DM2 (21 males and 12 females; mean age, 59.21 ± 12.60 yrs.; age range, 29-78 yrs) who had CMR examination were retrospectively enrolled in the study. The inclusion of CAD patient is according to the guideline of 2013 ESC guidelines on the management of stable coronary artery disease as the CAD is defined as an established pattern of angina pectoris, a history of myocardial infarction (MI), or the presence of plaque documented by catheterization [15]. In this research, all the CAD patients had plaque in the coronary arteries which were confirmed by X-ray coronary angiography. In those CAD patients, we screened out those who had DM2 defined as the diabetic CAD patients. The inclusions of DM2 were the International Diabetes Federation (IDF) in 2012. DM2 was clinically diagnosed according to the following criteria: 1) Fasting plasma glucose (FPG) ≥ 7.0 mmol/l (126 mg/dl); 2) 75 g oral glucose tolerance test (OGTT) with FPG ≥ 7.0 mmol/l (126 mg/dl) and/or 2 h plasma glucose ≥11.1 mmol/l (200 mg/dl); 3) Glycated hemoglobin (HbA1c) ≥ 6.5% /48 mmol/mol; 4) Random plasma glucose ≥11.1 mmol/l (200 mg/dl) in the presence of classic diabetes symptoms; 5) Asymptomatic individuals with a single abnormal test should have the test repeated to confirm the diagnosis unless the result is unequivocally elevated [16]. In addition, 18 normal subjects (12 males and 6 females; mean age, 49.78 ± 8.44 yrs.; age range, 35–65 yrs) were enrolled as normal control group during the same time of patients collecting. These normal control individuals were who underwent CMR for clinical suspicion of cardiovascular diseases but were confirmed as normal subjects by final clinical evidence. These individuals were confirmed no coronary stenosis by CMR coronary angiogram. Any individual who had chronic disease, hypertension (>140/90 mmHg), diabetes, family history of cardiovascular disease and diabetes, and arrhythmia or having ECG abnormality will not include in the normal control group. Written informed consent was obtained from all the patients and normal controls before the examination.

### CMR scanning

Cardiac magnetic resonance (CMR) was performed using a 3.0-T whole-body scanner (Trio Tim; Siemens Medical Solutions, Erlangen, Germany) in the supine position using ECG-triggering and the breath-hold technique. The dedicated cardiac phased array coil was utilized in all scanning procedures. Data acquisition was obtained during end-inspiratory breath holding. Localizing imaging, including coronal, sagittal and horizontal planes, were acquired by using the TrueFISP sequence (echo time 1.33 ms, repetition time 710 ms, flip angle 10°, slice thickness 8 mm, spacing between slices 24 mm, field of view 290 × 373 mm, and matrix size 146 × 224 mm). The short axis cine sequence was performed by using the Turboflash sequence (echo time 1.22 ms, repetition time 39.34 ms, flip angle 40°, slice thickness 8 mm, field of view 290 × 373 mm, and matrix size 146 × 224 mm). A total of 8–12 slices of short axis cine sequences were acquired in every subject from the level of the mitral valve to the level of the left ventricle apex. Left ventricular function and volume-time curve were measured on the short axis cine sequence. A four-chamber cine sequence (echo time 1.23 ms, repetition time 41.25 ms, flip angle 50°, slice thickness 8 mm, field of view 290 × 373 mm, and matrix size 146 × 224 mm) was obtained in one cardiac cycle. During the entire examination period, each subject’s condition was stable.

### Imaging analysis

**Fig. 1:**
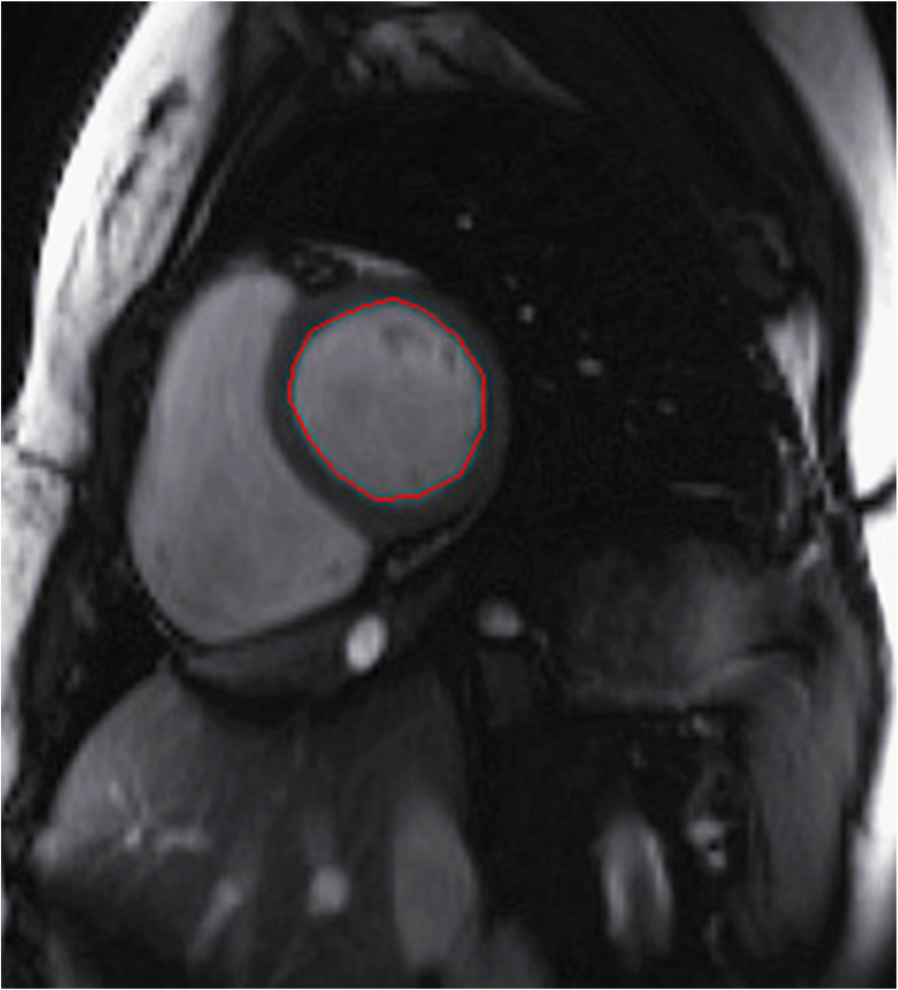
Endocardial boundary traced on the short-axis imaging by ARGUS to detect the volume-time curve

**Fig. 2:**
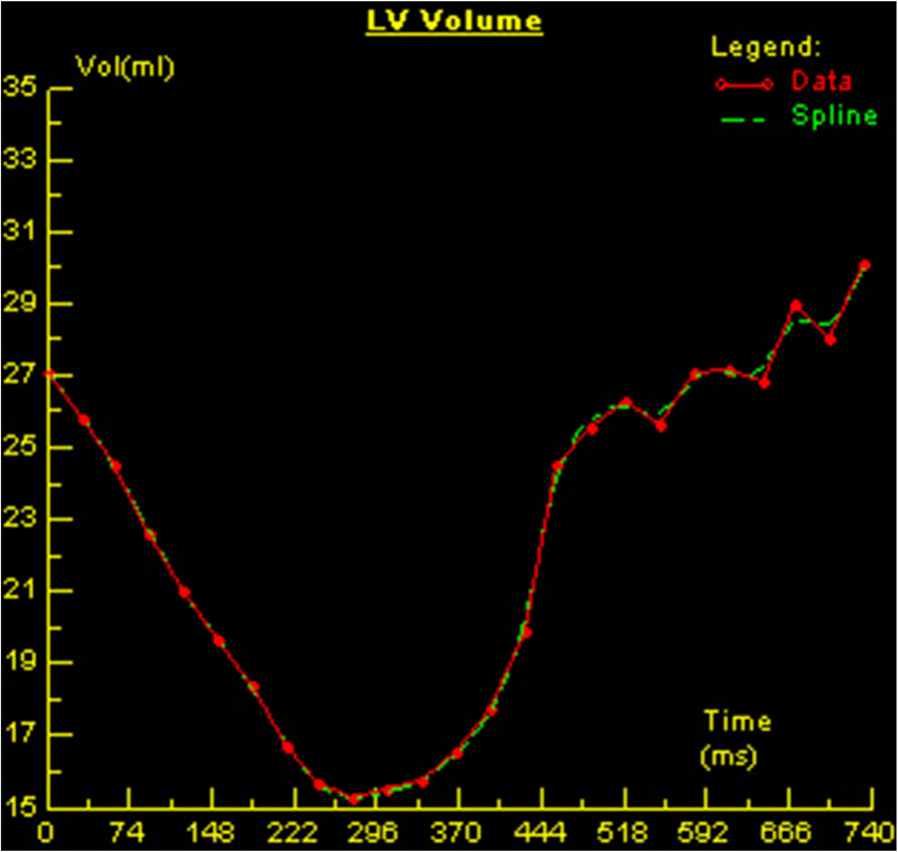
Volume-time curve of left ventricle

**Fig. 3:**
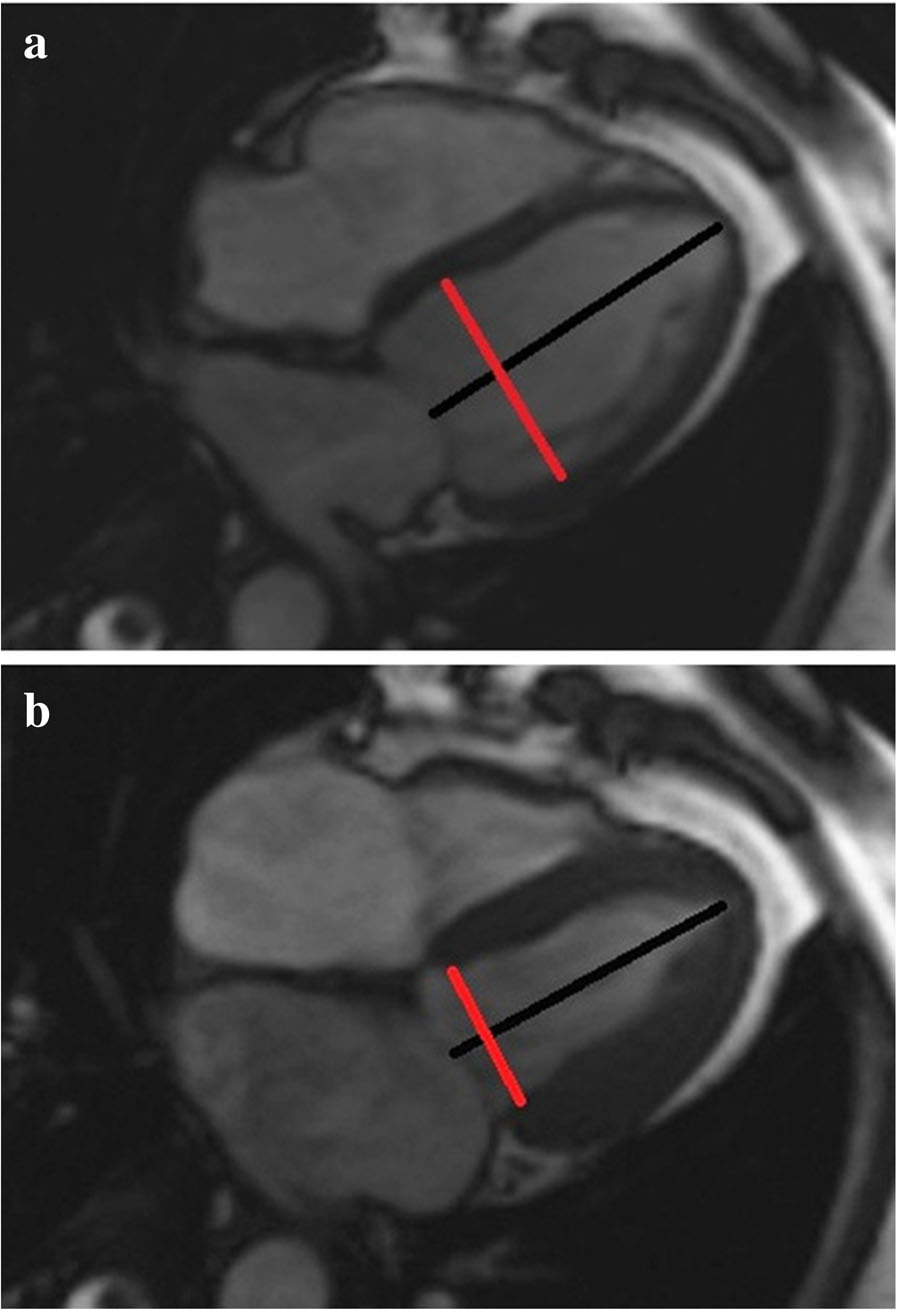
Measurement of morphologic parameters from four-chamber cardiac views. **a** LV end diastolic diameter. **b** LV end systolic diameter

An experienced radiologist analyzed the CMR data on the offline workstation (Leonardo workstation, Siemens, Erlangen, Germany). Global left ventricular function and the volume-time curve were analyzed on the short-axis cine images using offline post-processing software (Argus, Siemens Medical Solutions, Erlangen, Germany). Endocardial and epicardial borders were delineated manually from the image slices between the mitral valve and the apex at the end of diastole and at the end systole within the serial short-axis imaging; additionally, papillary muscle and moderator bands were carefully assigned to the cardiac lumen. Left ventricular function parameters (EDV, end diastolic volume; ESV, end systolic volume; SV, stroke volume; EF, ejection fraction and cardiac mass) were automatically obtained by Argus using Simpson’s method. The endocardial boundary of left ventricle was traced on every slice of the short-axis images (Fig. 1) and parameters of volume time curve (i.e., PER, PET, PFR, PFT, PER/EDV and PFR/EDV) were automatically obtained from software previously described (Fig. 2). The maximum LV diameter, including the end diastolic and end systolic diameters, were measured from the endocardium of the free wall to the interventricular septum on the four-chamber view; all diameter measurements were taken perpendicular to the long axis (Fig. 3). For the control subjects, we had performed the same imaging analysis as the patients group did by the same radiologist.

### Statistical analysis

Statistical analyses were performed with IBM SPSS (version 21.0, IBM SPSS Inc., Armonk, New York, US) and MedCalc (version 9.2.00). The data are presented as the mean ± standard deviation (SD). All data were checked for normality using the Kolmogorov-Smirnov test. All comparisons (Levene’s test) were assessed for and satisfied the homogeneity of variance assumption of the analysis of variance. The Levene’s test showed that *p* values of all data were greater than 0.05, which meant that all the data satisfied the hemogenetiy of variance. An independent *t*-test and non-parametric approaches (Chi-square test and Mann–Whitney U test) were utilized if appropriate to compare the basic characters between CAD with and without diabetes group. A one-way analysis (ANOVA) of variance with post hoc Bonferroni correction and Kruskal-Wallis were used to do the multiple comparisons (normal, CAD with and without diabetes groups) of the left ventricular functional, morphologic and volume-time curve parameters, ANOVA were used when the data conform to the homogeneity of variance and normal distribution and Kruskal-Wallis were used when the data is skewed distributions. Tukey’s range test has been used to conduct post-hoc analyses (after ANOVA) to verify the differences among the groups. Logistic regression was performed to screen out independent predictors of left ventricular dysfunction in CAD patients with diabetes. A receiver operating characteristic (ROC) was used to analyze the sensitivity and specificity of these predictors. A *p-*value <0.05 was considered significant for all statistical tests.

## Results

### Study population

A comparison of basic characteristics between the normal, CAD with DM2 and CAD without DM2 groups are presented in Table 1. The age, gender, heart rate and systolic/diastolic blood pressure were no significantly difference among the three groups all (all *P* > 0.05). The duration of DM2 in diabetic CAD patients ranged from 2 days to 14 years. The average HBA1c in CAD patients with diabetes was 9.38 ± 2.38%. Compared to non-diabetic CAD group, the plasma glucose of diabetic CAD patients was significantly increased (13.90 ± 6.38 mmol/L vs. 5.34 ± 1.65 mmol/L, *p* < 0.001). Electrocardiography revealed differences between diabetic and non-diabetic CAD groups with regard to pathological Q waves (6 [21.43%] vs. 9 [27.27%], respectively) and ST-T changes (20 [71.43%] vs. 32 [96.97%], respectively). Using catheter-based X-ray coronary angiography, 8 patients (28.57%) from the CAD with DM2 group and 13 patients (39.39%) from the CAD without DM2 group were found to have one-vessel coronary occlusion; two-vessel occlusion was found in 6 (21.43%) diabetic CAD patients and 4 (12.12%) non-diabetic CAD patients; and three-vessel occlusion was detected in 14 (50.00%) diabetic CAD patients and 16 (48.49%) non-diabetic CAD patients. All CAD patients, either with or without diabetes, had left ascending artery stenosis. 10 (35.71%) of the diabetic CAD and 17 (51.52%) of the non-diabetic CAD patients had left circumflex artery stenosis; 17 (60.71%) diabetic and 18 (54.55%) non-diabetic CAD patients were found to have right coronary artery stenosis. Percutaneous coronary intervention was performed in 21 (75.00%) diabetic CAD patients and 28 (84.84%) non-diabetic CAD patients.

**Table 1:** Basic characteristics of normal controls, CAD patients with diabetes and CAD patients without diabetes

	Normal (*n* = 18)	CAD without diabetes (*n* = 33)	CAD with diabetes (*n* = 28)	*p*
Age, years	49.78 ± 8.44	59.21 ± 12.60	58.12 ± 12.53	0.070
Male (n, %)	12 (66.66%)	21 (63.63%)	18 (64.28%)	0.976
HR, beats/min	82.06 ± 22.05	77.03 ± 12.43	81.19 ± 16.14	0.515
Systolic BP, mmHg	123.32 ± 22.13	127.81 ± 22.34	127.19 ± 23.40	0.800
Diastolic BP, mmHg	74.32 ± 12.10	74.24 ± 18.45	76.13 ± 18.41	0.449
Duration of diabetes			2 days to 14 years	N/A
Angina (n, %)	-	31 (93.94%)	16 (57.14%)	0.001
Cigarette Smoking (n, %)	-	21 (63.63%)	18 (64.29%)	0.958
Drinking (n, %)	-	16 (48.48%)	14 (50.00%)	0.906
Family history of cardiac disease (n, %)	-	4 (12.12%)	9 (32.14%)	0.057
Killips classification (I, II, III, IV)	-	27/4/2/0	23/3/2/0	0.991
Plasma Glucose (mmol/L)	-	5.34 ± 1.65	13.90 ± 6.38	<0.001
HBA1c,%	-		9.38 ± 2.38	N/A
Electrocardiogram changes (Pathologic Q waves) (n, %)	-	9 (27.27%)	6 (21.43%)	0.597
Electrocardiogram changes (ST changes) (n, %)	-	32 (96.97%)	20 (71.43%)	0.005
CMR LGE	-	33 (100%)	28 (100%)	N/A
Coronary artery stenosis (One/Two /Three-vessel)	-	13/4/16	8/6/14	0.635
Location of coronary artery occlusion (LAD/ LCX/ RCA)	-	33/17/18	28/10/17	0.641
CAD treatment (Drugs/ PCI/CABG)	-	5/28/0	7/21/0	0.335

The values reported are the mean ± SD

*CAD* coronary artery disease, *DM2* type 2 diabetes mellitus, *HR* Heart rate, *BP* blood pressure, *CMR* cardiac magnetic resonance, *LGE* late gadolinium enhancement, *LAD* left descending artery, *LCX* left circumflex artery, *RCA* right coronary artery, *PCI* percutaneous coronary intervention, *CABG* coronary artery bypass grafting

### Left ventricular function and size parameters

Left ventricular function and size parameters are shown in Table 2. Among the three groups, the diabetic CAD group had the lowest LVEF (all *p* < 0.05). The LVEDV of diabetic CAD patients was larger than the both the normal and non-diabetic CAD patients (all *p* < 0.05). LVEDD in the diabetic CAD group was significantly enlarged compared to both the normal and non-diabetic patients (5.44 ± 0.51 cm vs. 4.85 ± 0.34 cm, *p* < 0.001; 5.44 ± 0.51 cm vs. 5.13 ± 0.55 cm, *p* = 0.043). LVESD of both diabetic CAD and non-diabetic CAD patients were larger than normal controls (both *p* < 0.05).

**Table 2:** Baseline LV/RV function and morphologic parameters among normal, CAD patients with diabetes and CAD patients without diabetes

	Normal (*n* = 20)	CAD without diabetes (*n* = 33)	CAD with diabetes (*n* = 28)	*p*
LVEF,%	61.34 ± 4.48	45.29 ± 12.83*	41.9 ± 15.37*^#^	<0.001
LVEDV, ml	118.70 ± 23.69	147.14 ± 38.42*	163.22 ± 38.64*^#^	0.002
LVESV, ml	52.37 ± 23.72	83.15 ± 34.81*	98.26 ± 43.54*	0.001
LVSV, ml	75.11 ± 9.93	63.99 ± 16.63	64.96 ± 20.18	0.067
LVMASS, g	90.96 ± 22.57	130.17 ± 38.29*	139.99 ± 33.72*^#^	<0.001
LVEDD, cm	4.85 ± 0.34	5.13 ± 0.55*	5.44 ± 0.51*^#^	0.005
LVESD, cm	3.38 ± 0.36	3.79 ± 0.67*	3.98 ± 0.68*	0.017

*LV* left ventricle, *EF* ejection fraction, *EDV* end diastolic volume, *ESV* end systolic volume, *SV* stroke volume, *EDD* end diastolic diameter, *ESD* end systolic diameter

The values reported are the mean ± SD, *p* value of ANOVA were presented. *indicates *p*<0.05 compared to the normal group; ^#^indicates *p*<0.05 compared to the CAD without DM2 group.

### LV volume-time curve parameters

Volume-time curve parameters were calculated and presented in Table 3. Both the PER and PFR of CAD patients with diabetes were significantly decreased compared to normal controls and CAD patients without diabetes (all *p* < 0.05). PET was significantly different in all of the three groups, with the diabetic CAD group having the longest PET (*p* = 0.0008). PFT of diabetic CAD group was longer than both the normal group and non-diabetic CAD group (312.00 ± 143.25 msec in CAD with DM2 vs. 122.82 ± 37.64 msec in controls, *p* = 0.03; 312.00 ± 143.25 msec in CAD with DM2 vs. 147.92 ± 131.99 msec in CAD without DM2, *p* = 0.03). In comparing the PER/EDV and PFR/EDV values among the three groups, both the PER/EDV and PFR/EDV values were the lowest in the CAD with diabetes group, followed by the non-diabetic CADs, with the normal control group having the largest values; all comparisons were statistically significant (all *p* < 0.05).

**Table 3:** Comparison of the volume-time curve parameters of left ventricle

	Normal (*n* = 20)	CAD without diabetes (*n* = 33)	CAD with diabetes (*n* = 28)	*p*
PER (ml/s)	138.26 ± 25.04	118.59 ± 34.32*	90.35 ± 28.09*^#^	<0.001
PET (msec)&	113.52 ± 37.62	232.00 ± 215.10	336.74 ± 216.44	0.0008
PFR (ml/s)	169.55 ± 50.85	128.04 ± 37.35*	102.07 ± 36.11*^#^	<0.001
PFT (msec)	122.82 ± 37.64	147.92 ± 131.99	312.00 ± 143.25*^#^	0.004
PER/EDV (EDV/s)	5.44 ± 0.92	4.620 ± 1.21*	3.24 ± 1.12*^#^	<0.001
PFR/EDV (EDV/s)	6.48 ± 1.96	4.84 ± 0.99*	3.36 ± 1.06*^#^	<0.001

*PER* peak ejection rate, *PET* peak ejection time, *PFR* peak filling rate, *PFT* peak filling time from ES, *PER/EDV* peak ejection rate normalized to EDV, *PFT/EDV* peak filling rate normalized to EDV

The values reported are the mean ± SD, *p* value of ANOVA were presented. *indicates *p*<0.05 compared to the normal group; ^#^indicates *p*<0.05 compared to the CAD without DM2 group. & means the data is skewed distributions using Kruskal-Wallis analyzing

### Logistic regression analysis for independent predictors

All statistically significant left ventricular function, morphologic and volume-time index analyses were performed using logistic regression (Table 4). The odds ratio (OR) and 95% confidence interval for each of the parameters are shown in Table 4. The PET and PFT from the volume time curve were significant predictors of left ventricular dysfunction in diabetic CAD subjects (PET: OR 1.1208, 95% CI 1.0023 to 1.2532, *p* = 0.0454; and PFT: OR 1.0161, 95% CI 1.0001 to 1.0326, *p* = 0.0412). In addition, the PFR/EDV value was another independent predictor (OR 0.0139, 95% CI 0.0005 to 0.3955, *p* = 0.0123).

**Table 4:** Logistic regression of independent predictors of left ventricular dysfunction in CAD patients with diabetes

	OR	95% CI	*p*
LVEF	0.8494	0.6844 to 1.0541	0.1383
LVEDV	0.9355	0.8449 to 1.0358	0.1996
LVEDD	2.3366	0.0555 to 98.4616	0.6566
LVMASS	0.9991	0.9443 to 1.0572	0.9757
PER (ml/s)	0.9334	0.8014 to 1.0871	0.3756
PET (msec)	1.1208	1.0023 to 1.2532	0.0454*
PFR (ml/s)	0.9967	0.9894 to 1.0040	0.3736
PFT (msec)	1.0161	1.0001 to 1.0326	0.0412*
PER/EDV (EDV/s)	0.0917	0.0002 to 45.4775	0.4506
PFR/EDV (EDV/s)	0.0139	0.0005 to 0.3955	0.0123*

*OR* odds ratio, *CI* confidence interval

*p<0.05 in logistic regression analysis

### ROC analysis for predicting left ventricular dysfunction

**Fig. 4:**
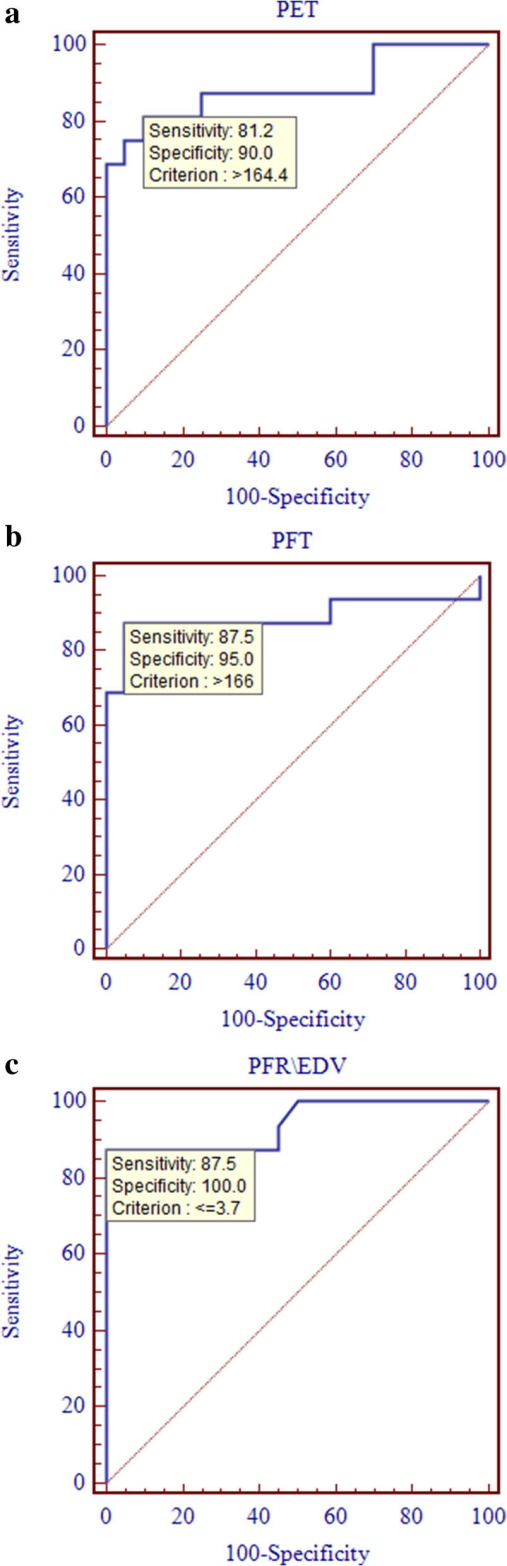
ROC analysis of PET, PFT and PFR/EDV in diabetic CAD subjects. **a** ROC analysis of PET. **b** ROC analysis of PFT. **c** ROC analysis of PFR/EDV

Regarding these independent predictors, the ROC curve of PET, PFT and PFR/EDV for CAD patients with diabetes was calculated and is shown in Fig. 4. The sensitivity and specificity of PET were 81.2 and 90%, respectively, using a criterion of greater than 164.4 msec (area under curve [AUC], 0.888; 95% CI, 0.737 to 0.967). The sensitivity and specificity of PFT were 87.5 and 95%, respectively, using a criterion of greater than 166.0 msec (AUC, 0.890; 95% CI, 0.741 to 0.969). The PFR/EDV value had a relatively high sensitivity (87.5%) and specificity (100.0%) using a criterion of less than 3.7EDV/s (AUC, 0.942; 95% CI, 0.810 to 0.991).

## Discussion

Several modalities can be used to evaluate left ventricular function, such as the SPECT and echocardiography [17]. However, both SPECT and echocardiography have some shortcomings; SPECT have invasive X-ray radiation and the spatial resolution of echocardiography is low, which may put an effect on the reliability and reproducibility of measuring the left ventricular function [18]. Cardiac magnetic resonance has become a accurate and reliable reference standard tool for evaluating left ventricular function due to its excellent spatial and temporal resolution, multiple parameters/plane and non-invasive characteristics [19]. Currently, the volume-time curve of CMR is considered to be an accurate tool for assessing cardiac function as a complement to conventional LV global function parameters like LVEF [20]. To define the volume-time curve index of CMR, the PER indicates the peak rate of left ventricular ejection, which is evaluated from the maximal LV ejection rate defined by the maximal change in the LV volume between sequential temporal phases (△volume/△phase). The PFR indicates the maximal LV filling rate defined by maximal change in LV volume between sequential temporal phases (△volume/△phase) [14]. PER/EDV represents the PER when normalized to EDV, and PFR/EDV represents the PFR when normalized to EDV. The PET is the time interval between the end of diastole and the PER, and the PFT is the time interval between the end of systole and the PFR. The PER, PET and PER/EDV are the parameters of systolic ejection function; the other parameters indicate diastolic filling function [20]. Type 2 diabetes mellitus is not only a metabolic disease but also an isolated risk factor of both cardiovascular and cerebrovascular disease, as demonstrated by previous studies; it may contribute to coronary artery disease and congestive heart failure. DM2 has an adverse effect on coronary microvascular dysfunction and left ventricular diastolic function. Previous research has found that DM2 patients had a lower PFR than normal individuals, indicating the presence of impaired myocardial relaxation and increased myocardial stiffness; consequently, the abnormal LV filling in DM2 patients is closely associated with abnormal myocardial perfusion [14]. In the study conducted by Zeidan et al., volume-time curves of CAD patients revealed significantly PFR [21]. Both DM2 and CAD are risk factors for alterations in volume-time curve parameters. In our study, the LV volume-time curve of diabetic CAD subjects was significantly changed alongside LVEF, LVEDV and LVEDD. The PER/EDV, PFR/EDV, PER and PFR values of diabetic CAD subjects were all reduced in comparison to non-diabetic CAD subjects and normal controls. However, PFT and PET were prolonged in the diabetic CAD group, demonstrating that the time interval between left ventricular filling and ejection in diabetic CAD patients was prolonged. In our study, the indexes that represent both diastolic filling (PFR, PFT and PFR/EDV) and systolic ejection (PER, PET and PER/EDV) dysfunction on the volume-time curve were reduced or prolonged in diabetic CAD subjects compared to non-diabetic CAD subjects. This may be explained by the notion that CAD in diabetic patients is not only a disease that affects epicardial vessels but also involves small vessel and myocardial damage [22]. Chronic hyperglycemia accelerates not only the formation of atherosclerotic plaques in coronary artery disease but also the deterioration of microvascular function as well as the automatic deregulation and interstitial fibrosis of the heart that is etiologic of left ventricular diastolic dysfunction [23–26]. Severe left ventricle dysfunction may be one reason why high mortality is seen in CAD patients with DM2 despite aggressive therapy [27]. In our research study, type 2 diabetes mellitus was an independent risk factor of left ventricular dysfunction in CAD patients with DM2. Because indices of conventional LV function, morphology and the volume-time curve were all changed in the diabetic CAD subjects in this study, logistic regression was performed to screen for independent predictors of left ventricular dysfunction. PET, PFT and PFR/EDV were found to be more significant than other LV function and size parameters. The ROC curve was calculated to detect the sensitivity and specificity of these predictors. The sensitivity and specificity of PET were 81.2 and 90%, respectively, utilizing a criterion of greater than 164.4 msec. The sensitivity and specificity of PFT were 87.5 and 95%, respectively, utilizing a criterion greater than 166.0 msec. The PFR/EDV value had relatively high sensitivity (87.5%) and specificity (100.0%), utilizing a criterion smaller than 3.7EDV/s. Since the type 2 diabetes mellitus, as an independent factor, can deteriorate the left ventricular function of CAD patients, which had been indicated in our and previous studies. Controlling well the plasma glucose of DM2 may postpone the heart failure of those CAD patients with type 2 diabetes mellitus. By the conventional LV functional measurement of our research, the left ventricular dysfunction of diabetic CAD was demonstrated, such as LVEF was decreased, and the LVEDV and LVEDD were increased in diabetic CAD patients compared to non-diabetic CAD patients; however, these indices were not significantly predicted by the logistic regression analysis. On the other hand, in this research, the PET, PFT and PFR/EDV values from CMR volume time curve were indicated to be valuable independent tools for with relatively high sensitivity and specificity detecting and predicting left ventricular dysfunction in diabetic CAD patients. LV volumetric quantification detection utilizing the volume-time curve may further elucidate differing degrees of left ventricle diastolic and systolic dysfunction between CAD patients with and without diabetes, beyond merely the ejection fraction, as previous researchers have reported [21]. All of the results of our research study indicate that the CMR volume-time curve may be a valuable tool for the assessment of left ventricular dysfunction in CAD patients with diabetes mellitus [28, 29].

We acknowledge a few limitations to this research study. First, a larger sample of diabetic CAD patients is needed to further investigate cardiac dysfunction, especially for assessing the diagnostic ability of the CMR volume time curve. Second, long-term follow up is necessary to determine the outcome and mortality differences between diabetic and non-diabetic patients; longitudinal data will help to illustrate the prognostic role of volume-time curve parameters with regard to heart failure and cardiac events in both study groups.

## Conclusions

In summary, our research has indicated that CAD patients with type 2 diabetes mellitus have more severe LV dysfunction, including diastolic filling and systolic ejection, compared with non-diabetic CAD patients. PET, PFT and PFR/EDV derived from the volume-time curve on cardiac MR can be used to predict left ventricular dysfunction in diabetic CAD patients with relatively high sensitivity and specificity.
